# Supplementary material for: Are carbon emissions trading and green financial instruments synergistic? -Comprehensive quantitative research based on content analysis
Source: PLoS One. 2024 Mar 7;19(3):e0298601. doi: 10.1371/journal.pone.0298601 (PMC10919601; doi:10.1371/journal.pone.0298601)
Supplement: S1 Data — (ZIP) [file pone.0298601.s001.zip › Data/地级市Z_F工作报告.zip!/╔╜╢1⁄2/╤╠╠¿╩╨/└┤╫╘╣1⁄2╓┌║┼í╛┬φ┐╦╩2╛▌═°í┐_2006.docx]

各位代表：

现在，我代表市人民政府，向大会作政府工作报告，请予审议，并请市政协委员和其他列席会议的同志提出意见。

一、进入新世纪，全市“十五”时期经济社会发展取得可喜成就“十五”计划是我市在新世纪实施的第一个五年计划。五年来，在上级党委、政府和中共烟台市委的正确领导下，全市人民坚持以邓小平理论和“三个代表”重要思想为指导，认真贯彻党的十六大和十六届三中、四中、五中全会精神，坚持以科学发展观统揽全局，全面落实省委、省政府“三个明显、两个高于、一个跨越”的发展要求和“东部突破烟台”的战略部署，认真执行市人大及其常委会的各项决议，聚精会神搞建设，一心一意谋发展，全市改革开放和现代化建设取得可喜成就。初步核算，2005年全市实现生产总值2012亿元，比2000年增加1133亿元，年均增长15.5%；人均生产总值30899元，增加17279元，按现行汇率计算达到3829美元，年均增长15.4%。境内财政总收入达到293亿元，增加188.5亿元，年均增长22.82%，其中地方财政收入87亿元，同口径年均增长29.73%。全社会固定资产投资完成1460.4亿元，增加1241亿元，年均增长46.6%。在省内和全国首批沿海开放城市中，我市主要经济指标多数实现位次前移，有5个县市区进入全国百强县和全省30个强县行列。

（一）经济结构调整跃上新台阶。五年来，全市上下大力调整经济结构，积极转变增长方式，产业素质明显提升，竞争力明显增强。一、二、三次产业增加值分别达到193.5亿元、1228.4亿元和590.6亿元，结构比例由2000年的14.4:51.8:33.8调整为9.6:61:29.4。纵深推进农业区域化布局、标准化生产、产业化经营，粮油、果品、蔬菜、畜牧、水产等优势产业已经形成，农业综合生产能力和比较效益明显提高。五大优势产业占农业增加值的比重达到96%，全市农产品出口达到17.7亿美元。大力推进北部沿海经济产业带和南部交通干线产业带建设，积极培植机械制造、电子信息、食品加工、黄金四大支柱产业和汽车、手机、电脑三大产品集群，初步形成了规模较大、层次较高、集群化发展的新型工业结构。2005年，全市规模以上工业企业完成销售收入3814亿元，年均增长32.8%；实现利税、利润分别达到415.6亿元和306.3亿元，年均增长32.9%和38.1%。全市销售收入过10亿元的企业达到47家，利税过亿元的企业60家，分别比2000年增加39家和49家。高新技术产业产值达到1120亿元，占规模以上工业比重由2000年的不足15%提高到28.6%。服务业增加值年均增长14.5%，重点行业发展迅速。2005年，全市社会消费品零售总额完成599.6亿元，年均增长13.2%。旅游业总收入达到117.5亿元，年均增长14.7%。全市银行机构期末本外币存款余额为1683亿元，其中城乡居民储蓄存款余额为1043亿元，分别比2000年底增加826亿元和452亿元。

（二）体制改革迈出新步伐。国有经济布局和结构的战略性调整取得明显成效，国有资产配置更加合理，监管体制基本建立，营运效益明显提高。市县乡村四级企业改制基本完成,现代企业制度普遍建立。民营经济快速发展，对全市经济社会发展的贡献份额进一步提高，2005年实现增加值和上缴税金所占比重分别达到79%和75%。农村税费改革扎实推进，全市农民减轻负担9.06亿元，从2005年起实现了农业“零税赋”。财税体制改革不断深化，财政用于公共服务、社会保障、农业等领域的支出持续增加。投融资体制改革进一步深入，投资主体多元化步伐明显加快。金融体制改革稳步推进。粮食流通体制改革率先完成。严格执行保护耕地的基本国策，国土资源市场化配置机制逐步完善，五年累计实现政府收益88.7亿元。住房货币化供应机制全面建立，廉租房和经济适用房建设规模不断扩大，五年共为困难群体提供经济适用房190万平方米、廉租房4.1万平方米。国家宏观调控政策得到有效落实，市场经济秩序明显改善。政府机构、行政审批、医疗卫生、市政公用事业、户籍制度等各项改革稳步推进。

（三）对外开放实现新跨越。积极应对入世带来的机遇和挑战，大力实施经济国际化战略，经济外向度、对外知名度明显提高。2005年全市合同利用外资65.06亿美元，实际利用外资25.5亿美元，其中外商直接投资19.08亿美元。五年累计实际利用外资60.4亿美元，是“九五”的4.24倍。2005年全市外贸进出口突破百亿美元大关，达到114.8亿美元，其中出口完成64.83亿美元，年均增长29.6%和26.9%。出口结构发生明显变化，高新技术产品和机电产品出口比重分别达到15.9%和34.9%，欧美市场对我市外贸出口的贡献率达到65.4%。各类园区健康发展，全市省级以上园区实际利用外资和外贸出口分别占全市总额的38%和42%。“走出去”战略进一步实施，对外承包、劳务输出和对外投资继续扩大， 对外友好往来不断加深。成功举办了APEC投资博览会和电子商务博览会、国际果蔬·食品博览会等一系列重大经贸活动，取得丰硕成果。

（四）城乡建设展现新面貌。围绕实现“年年有变化、三年见成效、五年大变样”的目标，按照“三高”要求，不断加大城市规划建设管理力度。五年全市完成城乡建设投资510亿元，其中中心城市完成300亿元。市中心区新建、扩建了滨海路、观海路、港城大街、山海路和机场路等30多条城市主次干道，建设了滨海景区、体育公园等一批重点项目。投资6亿多元对老城区48个社区进行了综合整治，受益居民13万户、40多万人。水电气暖等基础设施、市政公用设施更加完备，市区城市公共绿地面积达到1593.7公顷，比2000年增加967公顷。“拓展东西两翼，贯通南北山海”的城市发展战略初见成效，城市框架进一步拉开，整体形象明显提升。县级市和小城镇建设步伐加快，规模进一步扩大，功能日趋完善。全市城市化水平达到53%，比2000年提高7.2个百分点。荣膺“最佳中国魅力城市”称号，获得“中国人居环境奖”和“联合国人居奖”。大力推进城市基础设施建设，不断增强城市承载能力。五年全市累计完成交通基础设施投资160亿元。公路硬化里程10386公里，其中高速公路369公里，分别比2000年增加6749公里和193公里，实现了市市通有高速公路。蓝烟铁路复线建成通车，大莱龙铁路全线试运营。港口建设发展步伐加快，2005年，全市港口货物吞吐量达到8503万吨，集装箱吞吐量完成69.4万标箱，分别比2000年增加4847万吨和52.6万标箱，其中烟台港完成货物吞吐量4506万吨，集装箱吞吐量60万标箱，分别增加2940万吨和47万标箱。机场改造工程顺利实施，航线航班不断增加，2005年旅客吞吐量达到118万人次。烟大铁路轮渡、海阳核电、引黄调水等重大项目顺利推进。

（五）人民生活再上新水平。始终坚持以人为本的执政理念，把人民群众的利益放在首位，在不断增加群众收入的同时，注重解决群众在就业、就医、就学、住房等方面的实际问题，扎扎实实为群众办实事、办好事。2005年，全市城镇居民人均可支配收入12452元，农民人均纯收入5347元，分别比2000年增加5134元和1893元。全市物价基本稳定，居民消费价格总水平升幅控制在1.6%。五年新增城镇就业再就业45.5万人，城镇登记失业率控制在3.2%以内。全面建立起城镇职工养老、失业、医疗、工伤、生育“五位一体”的社会保险体系，农村养老保险覆盖面不断扩大，城乡低保家庭实现应保尽保，社会救助体系进一步完善。大力开展爱心捐助、送温暖、希望工程等公益活动，五年来共对困难学生、困难家庭和大病家庭发放爱心救助款1.42亿元，受益群众近42万人次。城市居民人均现住房使用面积和农民人均居住面积分别达到18.67平方米和27.66平方米，比2000年增加4.96平方米和1.54平方米。不断改善农村生产生活条件，强化农村公共服务。完成了农村电网改造工程、农村中小学危房改造工程，全市基本实现村村通公路、通客车、通电话，有居民的岛屿全部通航，深入开展了新型农村合作医疗制度试点，全面启动了村村通自来水工程。城乡居民恩格尔系数降到0.4以下。

（六）和谐社会建设取得新进展。全面实施科教兴烟和人才强市战略，全市五年累计取得各类科技成果820项，其中玉米新品种掖单13号的培育和推广、万吨油压双动铝挤压机两项成果获得国家科技进步一等奖，涌现出国内首例体细胞克隆牛、国家一类新药YH-16等自主创新成果。“产学研”合作机制逐步建立，国家、省级工程技术研究中心达到24个，高新技术企业发展到400家。义务教育普及率达到100%，高中段教育普及率达到88.7%，高等教育发展迅速，职业教育快速扩张。群众体育和竞技体育水平不断提高，在奥运会等国内外重大赛会上，我市运动员均取得优异成绩。文化卫生、新闻出版、广播电视、人口和计划生育、民族宗教、人民防空、民兵预备役、双拥共建、防灾减灾、史志档案、妇女儿童、残疾人及老龄等工作取得新成绩。基层民主建设不断加强，社会治安综合治理扎实有效，安全生产形势稳定。群众性精神文明创建活动深入开展，城乡文明程度显著提高。获得“全国文明城市”、“全国社会治安综合治理优秀城市”等荣誉称号。完成了对海阳市的帮扶并取得明显成效，启动了对栖霞市的帮扶工作，对定陶、聂拉木等地的对口帮扶工作均取得实质性进展。积极建设生态市和节约型社会，大力发展循环经济。万元生产总值耗能下降4.1%，主要污染物排放强度下降40%，城市空气质量良好率达到99%，全市森林覆盖率达到35%。

五年来，各级政府按照“为民、务实、清廉”的要求，着力加强自身建设，积极推进职能转变，切实精简行政审批事项，大力推行政务公开，行政效能进一步提高。决策目标、执行责任、考核监督“三个体系”不断完善，有效保障了决策落实。各级政府主动接受人大、政协及各民主党派、工商联和社会各界的工作监督、法律监督和民主监督。强化行政监察和审计监督，加强政风建设和公务员队伍建设，深入开展廉政教育和反腐败斗争，认真纠正部门和行业不正之风，严肃查处了一批违法违纪案件。

各位代表，五年的历程很不平凡。这五年是改革处于攻坚阶段、发展任务十分艰巨的五年，是区域竞争异常激烈、宏观环境并不宽松的五年，是我们战胜非典、禽流感疫情及其他重大自然灾害并取得全面胜利的五年。五年来，全市人民的思想观念发生了深刻变化，全市上下干事创业的氛围更加浓厚，加快发展的热情空前高涨。今天的烟台，经济健康发展，社会稳定和谐，人民安居乐业，充满生机和活力，同全国全省一样，站在了新的历史起点上！

各位代表，五年的探索和实践，使我们深深体会到，应对各种复杂形势和严峻挑战，实现经济社会发展的新跨越，必须始终坚持以邓小平理论和“三个代表”重要思想为指导，以加快发展为第一要务，以强市富民为第一目标，解放思想，加压奋进，求真务实，真抓实干；必须始终坚持以科学发展观统领全局，调整经济结构，转变增长方式，突出“五个统筹”，推动经济社会全面、协调、可持续发展；必须始终坚持深化改革、扩大开放，创新体制机制，增强发展的动力和活力，不断提升经济整体素质；必须始终坚持走烟台特色的发展路子，立足自身优势，理清发展思路，围绕事关全局和长远的重大战略和重大项目，集中用力，重点突破；必须始终坚持以人为本的执政理念和正确的政绩观，关心群众疾苦，维护群众合法权益，真心实意为人民群众办实事、做好事、解难题。我们坚信，只要坚持党的正确领导，坚持相信群众、依靠群众，全市上下团结一心、和衷共济，我们就一定能够不断夺取改革开放和现代化建设的新胜利。

各位代表，五年来，烟台的建设和发展得到了中央和省的极大关注和支持。胡锦涛总书记亲临烟台视察工作，省委、省政府三次在烟台召开现场会，先后对我们提出了“跨越发展”、“东部突破”、“走在前面”的殷切希望，给全市人民以极大的鼓舞和鞭策。我们深深感到，烟台所取得的一切成就，是上级党委、政府和中共烟台市委正确领导、科学决策的结果，是全市上下团结实干、顽强拼搏的结果，是社会各界和海内外友好人士热情参与、大力支持的结果。在此，我代表市人民政府，向全市广大工人、农民、知识分子、各级干部以及各阶层的建设者，向驻烟人民解放军、武警部队官兵和政法干警，向中央、省属驻烟单位，向各位人大代表和政协委员，向各民主党派、工商联、无党派人士和各社会团体，向所有关心支持烟台建设的港澳同胞、台湾同胞、海外侨胞和国际友人，致以崇高的敬意和衷心的感谢！

各位代表，五年来，我市经济建设和社会发展虽然取得了很大成就，但仍然存在许多矛盾和问题。一是经济发展的质量和效益还不够高。结构性矛盾仍然比较突出，服务业发展不快、比重较低，链条经济、产品集群、骨干企业培植还处于起步阶段。经济增长方式还没有根本转变，企业自主创新能力不强，资源使用效率较低，人才、能源和基础设施对赶超发展的支撑能力有待进一步增强。二是经济发展的机制体制性矛盾比较明显。市场机制还不完善、作用发挥得还不够好，改革攻坚的任务还十分艰巨。对外经贸的质量水平有待提高，应对国际贸易壁垒的能力还不够强。三是统筹发展、构建和谐社会面临较大压力。改善农村生产生活条件的任务还很繁重，农民收入增长不快，城乡弱势群体、特困阶层的困难解决得还不够好，社会保障体系有待进一步完善，社会事业发展相对滞后。四是政府职能和作风转变还没有到位。部分机关工作人员大局观念不强，服务意识淡薄，工作不扎实，办事效率不高；极少数人以权谋私，为政不廉，腐化堕落，败坏了政府形象。我们一定要正视这些问题，下决心认真加以解决。

二、站在新起点，奋力实现“十一五”发展的新突破

“十一五”时期是本世纪头20年重要战略机遇期的关键时期，具有承前启后的重要地位。我们一定要有高度的历史责任感、强烈的忧患意识和宽广的战略眼光，进一步解放思想，提升境界，抢抓机遇，开拓进取，站在新起点，实现新发展，开创新局面。

今后五年，我市经济社会发展的指导思想和总任务是：以邓小平理论和“三个代表”重要思想为指导，全面贯彻党的十六大和十六届三中、四中、五中全会精神，以科学发展观统揽全局，以“又快又好、赶超发展、走在前面”为目标，以经济结构调整和转变增长方式为主线，以建设制造业强市、增强自主创新能力、建设社会主义新农村和构建和谐社会为重点，大力实施经济国际化、城市化、以港兴市和科教人才强市战略，进一步调动和凝聚全市上下的力量，推动国民经济持续快速健康发展和社会全面进步，全面建成小康社会，为提前基本实现现代化奠定坚实基础。

“十一五”期间，全市国民经济和社会发展要努力实现“六个新突破”。

（一）坚持科学发展、赶超发展，加快经济结构战略性调整，积极转变经济增长方式，增强自主创新能力，努力实现经济综合实力的新突破。加速膨胀机械、电子、食品、黄金四大支柱产业，积极发展石化、冶金、生物制药三大潜力产业，集中培育汽车及零部件、船舶及配套产品、电脑、手机、葡萄酒、金银珠宝六大产品集群，打造现代制造业基地，构建富有竞争力的现代产业体系。繁荣发展生活性服务业，创新发展生产性服务业，积极发展新型服务业，努力扩大服务业规模。大力实施科教人才强市战略，鼓励原始创新，引导集成创新，大力推动引进消化吸收再创新，加快发展高新技术产业，提高国民经济和社会信息化水平。到2010年，全市生产总值达到3700亿元，年均增长13%，人均达到6800美元。三次产业结构比例调整为6:61:33,高新技术产业产值占规模以上工业比重提高到38%。地方财政收入达到200亿元，年均增长20%。

（二）坚持城乡统筹发展，推进工业反哺农业、城市支持农村，着力抓好“三农”工作，努力实现建设社会主义新农村的新突破。按照生产发展、生活宽裕、乡风文明、村容整洁、管理民主的目标和要求，大力发展农村经济，积极推进农业结构调整，繁荣发展农村二、三产业，实现农村经济与城市经济的互促互动，促进农民持续增收。加大政府对农村公共服务投入，强化农村基础设施建设，优化农村生态环境，改善农民生产生活条件。切实提高农村科技、教育水平，推进农村文化建设。切实抓好农村基层组织建设，扩大农村基层民主，保障农民依法行使民主权利。到2010年，全面实现农村免费义务教育，全面推广农村新型合作医疗，全面实现农村最低生活保障，全面推广农村养老保险，全面建立农民培训就业体系，加快构筑新型城乡关系。

（三）突出中心城市地位，进一步拉开框架、丰富内涵，完善城镇体系，努力实现城市化的新突破。全市城市发展要以建设现代化国际性港口城市为目标，按照“两带、两轴”，即“北部滨海城市带、南部滨海发展带、烟-青发展轴、龙-莱-海发展轴”的总体格局展开。中心城市继续按照“拓展东西两翼、贯通南北山海、适度向南发展”的思路，着力建设以“生态、人文、山海”为特色的富有魅力的滨海城市。县域中心城市要着力增强产业支撑和辐射带动能力，加快膨胀经济规模和城市规模。积极推进有一定实力和规模的中心镇向大中城市“卫星城”、各具特色“小城市”方向发展。到2010年，中心城市人口达到200万人，7个县级城市全部进入中等城市行列，中心镇和重点镇人口分别达到3-5万人，全市城市化水平达到60%。初步形成中心城市功能完善、县域城市协调发展、农村城镇化水平较高的新型城市体系。

（四）大力推进市场取向的各项改革，加快构建适应WTO规则、互利共赢的开放格局，努力实现改革开放的新突破。更大程度地发挥市场在资源配置中的基础性作用，消除影响科学发展的体制障碍，使各种经济成分更具活力，各类市场主体更具创造力，区域经济发展更具竞争力。

充分发挥区位优势，统筹国际、国内两个市场、两种资源，主动承接国际产业和国际资本转移，努力提高利用外资质量和水平，大力转变外贸增长方式，积极实施“走出去”战略，实现产业结构与国际对接、投资环境与国际接轨。到2010年，全市外贸出口总额达到130亿美元，年均增长15%。实际利用外商直接投资五年累计达到128亿美元。

（五）加快推进重大基础设施建设，培养和引进优秀建设人才，努力实现赶超发展支撑条件的新突破。大力实施“以港兴市”战略，全面启动烟台港西港区建设，着力构建以烟台港为龙头、龙口港为骨干、其他中小港口为补充的港口群。到2010年，全市港口货物吞吐量达到2亿吨，集装箱达到200万标箱。建设龙烟铁路、青（岛）烟（台）荣（成）城际铁路烟台段，加快烟台机场扩建改造，构建“五纵五横一环”的公路干道格局，形成现代化、综合性陆海空立体交通体系，提高烟台在东北亚、环渤海经济圈和国家南北大通道中交通枢纽城市的地位。抓好海阳核电、引黄调水等项目建设，强化能源水利保障。全社会固定资产投资五年累计完成11000亿元，年均增长18%。积极构筑人才和科技高地。到2010年，全市高层次人才达到5万人，高新技术企业达到500家。

（六）坚持以人为本，全面建成小康社会，进一步提升全国文明城市、最安全城市的层次和水平，努力实现构建社会主义和谐社会的新突破。加快发展各项社会事业，着力解决好事关民生的重大问题。努力增加就业再就业，五年内，全市新增城镇就业再就业50万人，登记失业率控制在3.5%以内。进一步健全完善社会保障体系，扩大社会保障覆盖面。建立公平的收入分配机制和教育、医疗、文化服务体系，不断扩大公共服务惠及面，努力提高城乡居民收入。到2010年，全市城镇居民人均可支配收入达到19100元，农民人均纯收入达到7800元，年均分别增长9%和8%。扎实推进“最安全城市”和“诚信烟台”建设。发扬社会主义民主，完善社会主义法制，促进物质文明、政治文明、精神文明建设全面进步。加快建设生态市，积极构建资源节约型和环境友好型社会，促进人与自然的和谐。到2010年，万元GDP耗能降低到0.75吨标准煤、耗水降低到75吨，森林覆盖率达到40%。

各位代表，关于未来五年发展的具体任务目标和工作重点，《烟台市国民经济和社会发展第十一个五年总体规划纲要（草案）》做出了全面安排。《纲要（草案）》已提交大会，请予审议。

三、瞄准新目标，确保“十一五”开好局、起好步

2006年是实施“十一五”规划的开局之年，做好今年的政府工作，意义十分重大。我们要进一步转变发展观念，创新发展模式，提高发展质量，推动经济社会切实转入科学发展的轨道，努力实现又快又好发展。今年经济社会发展的主要预期目标是：生产总值增长14%，全社会固定资产投资增长20%，社会消费品零售总额增长16%，外贸出口增长17%，实际利用外商直接投资增长10%，地方财政收入增长20%，城镇居民人均可支配收入增长10%，农民人均纯收入增长8%，城镇登记失业率控制在3.4%以内，人口自然增长率控制在2‰左右。在实际工作中要力争更快更好一些。

（一）进一步做好“三农”工作，加快社会主义新农村建设。一是加快发展现代农业。以农民增收为中心，突出发展优质、高产、高效、生态、安全农业，积极推进农业和农村经济结构调整，稳定粮油生产，优化发展种植业，做大做强畜牧业，巩固提高水产业。搞好农业新技术推广应用，提高农业机械化水平，不断增强农业综合生产能力。继续开展“中国食品名城”创建活动，搞好农村新型合作组织建设，抓好“百强”龙头企业，推进农业标准化生产。大力发展非农产业，继续实施农民科技培训工程、农村劳动力转移培训阳光工程、新型农民创业培植工程，促进农村劳动力向城镇和二、三产业转移。二是加大对“三农”的投入。对“三农”的扶持由主要靠“少取”转向“多予、放活”，今年安排6800万元财政资金，支持农村基础设施和公益设施建设。继续严格落实国家和省各项支农惠农政策，加大市县财政对农业和农村的支持力度，逐步缩小城乡之间人均公共服务和生活水平差距。切实强化农村“路水电医学”等设施建设，巩固村村通油路成果，推进有线电视村村通、户户通，大力实施村村通自来水工程，确保今年农村自来水普及率达到75%以上。扩大新型农村合作医疗试点范围，今年全市新型农村合作医疗覆盖面达到85%以上。集中开展村容村貌整治活动，加快解决农村脏乱差问题。三是推进农村文明建设。提高农民思想道德素质和文明守法意识，完善村民自治机制，扩大基层民主。搞好“三下乡”活动，积极倡导健康、文明、科学的生活方式，创造农村精神生活丰富、人与人和谐相处、安居乐业的良好环境。建设社会主义新农村的难点在弱势村。今年要组织发动企业、机关、小康村结对帮扶弱势村，开展“三加一”联手共建社会主义新农村活动。

（二）坚持走新型工业化道路，着力打造制造业强市。以科技进步和自主创新为先导，着力培育制造业发展的规模优势、技术优势和品牌优势，促进全市工业结构优化升级。全面启动实施以抓大培强为主要内容的“三个五十”工程，集中培育50户重点骨干企业，争创50个名牌产品，建立50家具有较强自主创新能力的技术中心。一是培育壮大优势产业和龙头企业。以四大支柱产业和三大潜力产业为重点，聚集优势资源，加强政策扶持，加快企业产权多元化改革步伐，搞好与国外大公司、大商社的对接，促进优势骨干企业快速膨胀，培育拉动全市经济快速发展的新龙头。二是全面增强企业创新能力。以电子信息、新材料、生物制药、先进制造等领域为重点，加大科技投入，加强应用技术和高新技术研究，注重搞好自主知识产权的开发和保护。抓好东岳动力总成第三代发动机、浪潮乐金扩产等一批重点技改项目，加快国家一类新药YH-16、芳纶1414等一批重大科技成果的产业化步伐。年内，全市力争完成技改投资480亿元，增长25%；创建市级技术中心20家以上，省级技术中心4家。三是加大品牌培植力度。积极实施名牌带动战略，引导和支持优势骨干企业加强品牌经营，放大品牌效应，叫响“烟台制造”、“烟台创造”，力争有更多的优势产品获得中国名牌和全国驰名商标称号。

（三）加快发展现代服务业，进一步提升产业层次。坚持膨胀规模和优化结构并举，推进服务业发展与现代工业发展相结合，与加快城市化进程相结合，与扩大内需相结合，带动服务业结构优化升级。一是做大做强商贸物流餐饮业。扎实推进流通行业“三个十工程”建设，加快培植一批大型骨干流通企业和市场，规划建设一批物流园区及现代物流设施，积极发展连锁经营、物流配送、电子商务、现代批发市场等新型流通业态。发展商务服务，引进和扶持大众餐饮品牌。进一步扩大农村市场，抓好“万村千乡”市场工程试点，引导便利店和超市向农村延伸。二是快速扩张旅游会展业。加大旅游资源开发整合力度，培育中心城市和“蓬长龙”旅游强势板块，加快滨海生态旅游目的地规划建设，打造“山海仙境·葡萄酒城-魅力烟台”的城市形象品牌。办好亚欧会议旅游合作发展论坛暨展览会、第八届国际果蔬·食品博览会和中国国际美食节。三是加快发展金融业。创新金融服务方式，开发新的金融产品，推动银企合作。积极培育资本市场，整合开发金融资源，健全完善金融协调工作机制，优化金融生态环境，进一步提升金融产业化发展水平。四是突破发展文化传媒业。完善文化产业发展政策，扶持培育一批大型文化企业集团。开工建设市京剧院新院、市群众艺术馆新馆等文化设施。同时，积极发展中介服务、社区服务和房地产等服务业，构建服务业发展的新格局。优化服务业发展环境。进一步打破产业政策分割，降低市场准入门槛，规范各种收费行为，理顺水电等价格；进一步打破所有制分割，鼓励支持外资和有实力的民营企业、社会法人参与兴办服务业项目。

（四）进一步提高对外开放水平，形成全方位、宽领域、多层次的开放格局。一是着力提高利用外资质量。进一步强化产业招商和专业招商，积极引进国外大公司、大商社的资金、技术和研发机构。坚持引资区域多元化，巩固扩大对日韩和港澳台地区的招商，着力引进欧美高科技项目和资金技术密集型项目，努力拓展与东盟、印度等地的合资合作。进一步强化园区和企业的招商主体作用，提高园区投资强度和项目密集度，鼓励有条件的企业加强与国外知名企业的合资合作，支持现有外资企业增资扩股。二是大力转变外贸增长方式。坚持科技强贸、以质取胜，巩固扩大农副产品、纺织服装出口规模，提高机电和高新技术产品出口比重。积极承接国外制造业转移和服务外包，注重提高加工贸易质量和水平，促进加工贸易转型升级，努力打造自主出口品牌。扩大紧缺资源性产品进口，引进国外先进设备和管理方式。高度重视解决贸易争端和摩擦，提高反倾销、反贸易壁垒应对能力。三是加强国际经济技术合作。积极实施“走出去”战略，扶持和引导企业境外投资，促进我市产品出口和境外资源利用。支持企业对外承包工程，扩大劳务输出规模。四是进一步强化区域经济合作。突出抓好与国内企业500强、民营企业500强和上市公司的联系对接，推动我市大企业与国内大公司的战略合作。巩固与“珠三角”、“长三角”地区的合作交流机制，促进与京津地区及环渤海经济圈有关城市的经济合作与交流，扩大与东北老工业基地和西部地区的经贸往来。

（五）全面深化各项改革，完善赶超发展的体制保障。一是深化行政管理体制改革。积极转变政府职能，加快推进政企分开、政资分开、政事分开、政府与市场中介组织分开，强化社会管理和公共服务职能，减少和规范行政审批，推行电子政务，提高行政效率，降低行政成本。二是深化企业改革。加快推进产权制度改革，健全现代企业制度，完善国有资产监管运营体系，促进国有资产保值增值。深化垄断行业和公用事业改革，推进投资主体多元化。年内要完成2-3户市管企业的投资主体多元化改造，完成40户以上企业的改革改制，基本完成上市公司的股权分置改革，推动更多企业到境内外资本市场上市。进一步放宽非公有制经济准入条件，依法保护非公有制经济合法权益和私有财产，鼓励发展各具特色的专业园区、专业乡镇和专业村，大力培育民营高新技术企业和龙头骨干企业，推动民营经济上规模、上水平。三是深化农村经济体制改革。以乡镇机构、农村义务教育和县乡财政体制等配套改革为重点，进一步深化农村税费改革，做好村级债务化解工作，防止农民负担反弹。严格执行国家和省土地承包政策，稳定土地承包关系，促进土地合理流转。四是深化事业单位改革。规范精简行政支持类事业单位，放权搞活社会公益类事业单位，推动经营开发服务类事业单位进入市场，逐步建立与市场经济体制相适应的政事职责分开、法人地位明确、单位自主运营、政府依法管理的新型管理体制。五是深化综合配套改革。加强税收征管，积极培植财源。坚持公共财政改革取向，继续完善市对县的财税管理体制，稳步推进国库集中支付制度改革，加大公共服务和社会公益事业投入。深化投融资体制改革，健全政府投资决策责任制。改进价格管理方式，规范市场价格秩序。完善粮食储备和管理体系，增强政府调控能力。

（六）加快城市现代化进程，带动区域经济协调发展。一是加快推进城市基础设施建设。年内重点抓好烟台港西港区、龙口港屺坶岛港区、国际机场飞行区改扩建工程，加快海阳核电、引黄调水、龙烟铁路龙口至蓬莱段、国道206线蓬莱至龙口段等项目建设进度，确保烟大铁路轮渡投入运营。做好青烟荣城际铁路项目和青岛至龙口高速公路项目的前期工作。新建扩建红旗东西路隧道、学院路、幸福南路、化工路等市区道路，改造完善滨海景观带、港站广场和文化广场，加快推进套子湾污水处理厂二级处理工程、辛安河污水处理厂扩建工程和垃圾处理厂扩建工程，确保完成体育公园二期和会展中心建设工程。抓好北方温州商贸城、国际产业转移配套社区、雨岱山旅游开发等市区服务业重点项目建设。二是继续加大老城区改造力度。大力实施“洁绿亮美”工程，坚持整治与开发相结合，抓好幸福、凤凰台、白石等重点区片改造，完成朝阳街沿海部分改造工程，改善市民居住环境，着力提升宜居城市水平。三是加强城市规划与管理。不断完善城市规划体系，强化城市规划的严肃性和约束力，保持规划与实施的一致性。改进市区管理体制，健全城市管理行政执法体系，推进中心区网格管理试点。四是大力发展县域经济。进一步扩大县级经济管理权限，强化对县域经济的政策扶持、规划引导、产业指导和调度考核。依托北部沿海经济产业带和南部交通干线产业带建设，带动各县市区调整产业结构，发展特色经济，壮大优势产业，推动更多的县市进入全国百强县和全省强县行列，已经进入的争取位次前移。继续实施促强扶弱战略，抓好帮扶栖霞工作。鼓励各县市区之间开展多种形式的区域经济协作和技术、人才合作，推动生产要素在区域间的自由流动和优化配置。

（七）增强自主创新能力，统筹推进各项社会事业。积极建设创新型城市，努力构建新型社会事业发展体系，促进经济社会协调发展。一是大力推进自主创新。深化科技体制改革，建立完善政府支持、企业主导、“产学研”相结合的科技创新体系。实行有利于自主创新的财政、人才等政策，坚持引进技术和引进智力并举，努力培育自主知识产权。加强重点实验室、工程技术研究中心、中试基地建设，提高科技研发能力。积极开展“科技自主创新年”活动，加大对汽车及关键零部件、船舶制造装备技术、电子电器关键技术、新型材料技术、生物制药技术等重大科技项目的研发投入。广泛运用信息技术改造传统产业，促进信息技术普及应用，推动基础性和公益性信息资源的开发、整合与共享，着力打造“数字烟台”。二是加强人才培养和开发。牢固树立人才是第一资源的观念，调整优化人才队伍结构，加强人才引进工作，建立健全分级分类培训教育体系。进一步深化选人用人制度改革，完善有利于优秀人才脱颖而出的用人机制。三是优先发展教育事业。切实抓好基础教育，巩固提高义务教育成果。提高高中段教育普及率，继续扩大优质高中教育资源覆盖面。大力发展高等教育，高度重视职业教育。抓好中国农业大学烟台校区一期、烟台职业学院扩建、技术学院新校等建设。实施“十万技工培养计划”和“金蓝领”培训工程。鼓励发展民办教育，运用市场手段和产业运作方式，广泛吸纳社会资金发展教育事业。四是促进社会事业全面进步。实施文艺精品工程，繁荣发展文化事业。立足建设体育强市，大力实施“全民健身计划”，鼓励社会力量参与体育市场开发。精心组织承办好第21届省运会。全面实施食品药品安全工程，调整优化卫生资源，加大药品价格和医疗收费监管力度，努力解决群众看病难、看病贵的问题。扩大就业总量，提高就业质量，积极为困难群众提供就业援助。年内确保新增城镇就业再就业12万人以上，其中失业人员再就业3.5万人以上。巩固完善“五位一体”的城镇保险体系，依法加大社会保险扩面征缴工作力度，健全农村养老保险制度。进一步健全社会救助体系，鼓励发展社会慈善事业，切实保障困难群众的基本生活。做好人口和计划生育工作，加快推进农村部分计划生育家庭奖励扶助制度试点。积极发展妇女儿童、残疾人和老龄事业，大力发展新闻出版事业，推进广播电视数字化，依法搞好新一轮地方史志修编工作，抓好外事侨务、气象地震、民族宗教工作，加强国防教育和国防动员，加大人民防空与民兵预备役建设力度，争创全国全省双拥模范城。

（八）加快构建社会主义和谐社会，不断提升城市现代文明水平。一是加强生态环境保护，建设生态城市。从严控制工业污染排放，实施流域污染综合治理，加快推进海洋生态整治。大力开展植树造林活动，集中突破城区和荒山绿化，积极推进荒山和宜林地产权改革，鼓励各方面力量参与造林开发。二是大力发展循环经济，建设节约型城市。加大节能执法力度，搞好新能源和可再生能源的开发利用，推进城市生活垃圾减量化、资源化、无害化。全面推进节约用水，加快发展节水型农业、旱作农业，加强工业用水定额管理，大力推行中水回用。合理开发利用资源，深入扎实地搞好土地和矿业秩序整顿。提高全民节约意识，建立绿色消费体系，推进政府机关和公共设施节能工程，提倡购买使用再生用品和环保产品。三是营造安定团结的社会局面，建设平安城市。建立健全应急管理和应急救援体系，提高保障公共安全和处置突发事件的能力，提高各种自然灾害的预测、预防和预警能力。加强安全生产监管，改善安全生产条件，遏制重特大事故发生。深入开展严打整治斗争，落实社会治安综合治理措施，切实保护人民生命财产安全。全面推进“五五”普法依法治理工作，进一步提高全民法律素质和全社会法治化管理水平。认真贯彻落实《信访条例》，解决好涉及群众切身利益的各种问题，化解社会矛盾，维护社会稳定。四是促进政治文明和精神文明，建设文明城市。进一步完善城乡基层政权、基层自治组织、企事业单位民主管理制度，积极推行政务公开、厂务公开和村务公开。深化市民思想道德建设，广泛开展爱国主义、集体主义、社会主义教育，突出抓好社会公德、职业道德和家庭美德建设。构建社会信用体系，打造“诚信烟台”。开展“讲文明、改陋习、树新风”活动，提升市民素质，巩固提高全国文明城市建设成果。

四、迎接新挑战，切实加强政府自身建设

组织实施好“十一五”规划，做好今年的工作，把改革开放和现代化建设不断推向前进，必须不断加强政府自身建设，努力打造“创新、务实、法治、为民、廉洁”政府，不辜负党和人民的重托。

（一）坚持解放思想，努力建设创新政府。要进一步加深对邓小平理论、“三个代表”重要思想和科学发展观的学习理解，努力掌握市场经济知识和现代科学文化知识，不断提高理论素养、知识水平和创新能力。要善于把握世界经济发展的新规律，善于把握科技发展的新趋势，善于把握改革开放的新形势，善于把握加快发展的新机遇，不断提升思想境界，努力在更高层次上谋划全局、推动发展。要注重吸收先进地区的新理念、新做法，注重发掘人民群众在实践中创造出的新经验、新典型，认真总结，大胆实践，不断创新。

（二）坚持实事求是，努力建设务实政府。树立正确的政绩观，弘扬埋头苦干、求真务实的优良作风，弘扬规范严谨、周密细致的科学精神，不搞不切实际的“形象工程”、“政绩工程”，不搞好大喜功的虚报浮夸、盲目攀比，做到说实话、办实事、求实效。进一步完善和落实决策目标、执行责任、考核监督“三个体系”，提高各级政府抓大事、抓落实的能力，确保政令畅通。进一步转变政府职能，把工作重心转到经济调节、市场监管、社会管理和公共服务上来，努力提高行政效率和服务水平。

（三）坚持依法行政，努力建设法治政府。认真贯彻依法治国的基本方略，全面实施《公务员法》和《行政许可法》等法律法规，严格依照法定权限和程序行使权力，履行职责，接受监督。推进行政执法责任制，对行政过错要依法追究，建立起权责统一的管理机制。积极推进政务公开，进一步完善重大事项公示制度和听证制度，保证人民群众依法行使知情权、参与权和监督权，推进行政决策的科学化、民主化。坚持向人大及其常委会报告工作和向人民政协通报情况的制度，自觉接受市人大及其常委会的法律监督，市政协、各民主党派、工商联、党外人士和各社会团体的民主监督，广大人民群众的监督和新闻舆论监督，广开参政议政渠道，齐心协力推进社会主义现代化建设。

（四）坚持以人为本，努力建设为民政府。牢固树立以人为本、执政为民的理念，深怀爱民之心，恪守为民之责，善谋富民之策，多办利民之事，克服官僚主义、形式主义，深入基层，深入群众，体恤民情，了解民意，始终保持与人民群众的血肉联系。要以扩大就业、卫生医疗、社会保障、扶贫解困和保障安全等工作为重点，继续实施为民服务实事工程，切实解决好人民群众最关心、最直接、最现实的问题。要尊重劳动、尊重知识、尊重人才、尊重创造，积极为人民群众干事创业、发挥特长搭建平台，为实现人的全面发展创造良好环境。

（五）坚持艰苦奋斗，努力建设廉洁政府。各级政府要把廉政建设和反腐败斗争作为一项重大政治任务抓紧抓实抓好。坚持标本兼治、综合治理，惩防并举、注重预防，抓紧建立健全与新形势、新环境、新任务相适应的教育、制度、监督并重的预防和惩治腐败体系，从源头上预防和治理腐败，坚决纠正损害群众利益的不正之风。要教育各级公务员严于律己，克己奉公，清白清廉做人，公平公正做事。要重点加大对各级领导干部和权力部门、重点行业的监督力度，从严防范和惩治公务人员失职渎职行为和职务犯罪行为，以反腐倡廉的实际成果取信于民。对腐败行为和腐败分子要依法依纪，严肃追究，严惩不贷。

各位代表，站在新起点、实现新发展、开创新局面，是人民寄予的历史重托，是时代赋予的庄严使命。让我们更加紧密地团结在以胡锦涛同志为总书记的党中央周围，高举邓小平理论和“三个代表”重要思想伟大旗帜，全面落实科学发展观，在中共烟台市委的坚强领导下，振奋精神，锐意进取，开拓创新，扎实工作，为开创“十一五”时期经济社会发展的新局面而努力奋斗！
